# What is an expert? A systems perspective on expertise

**DOI:** 10.1002/ece3.926

**Published:** 2013-12-26

**Authors:** Michael Julian Caley, Rebecca A O'Leary, Rebecca Fisher, Samantha Low-Choy, Sandra Johnson, Kerrie Mengersen

**Affiliations:** 1 Australian Institute of Marine SciencePMB 3, Townsville, Qld, 4810, Australia; 2 Australian Institute of Marine Science, UWA Oceans InstituteCrawley, WA, 6009, Australia; 3 School of Mathematical Sciences, Queensland University of TechnologyGPO Box 2434, Brisbane, Qld, 4001, Australia; Rebecca A. O'Leary, Department of Agriculture and Food, Western Australia3 Baron-Hay Court, South Perth, WA, 6151, Australia

**Keywords:** Bayesian network, expert judgement, expert knowledge, expert opinion, hierarchy of classes, supra-expert, taxonomy

## Abstract

Expert knowledge is a valuable source of information with a wide range of research applications. Despite the recent advances in defining expert knowledge, little attention has been given to how to view expertise as a system of interacting contributory factors for quantifying an individual's expertise. We present a systems approach to expertise that accounts for many contributing factors and their inter-relationships and allows quantification of an individual's expertise. A Bayesian network (BN) was chosen for this purpose. For illustration, we focused on taxonomic expertise. The model structure was developed in consultation with taxonomists. The relative importance of the factors within the network was determined by a second set of taxonomists (supra-experts) who also provided validation of the model structure. Model performance was assessed by applying the model to hypothetical career states of taxonomists designed to incorporate known differences in career states for model testing. The resulting BN model consisted of 18 primary nodes feeding through one to three higher-order nodes before converging on the target node (Taxonomic Expert). There was strong consistency among node weights provided by the supra-experts for some nodes, but not others. The higher-order nodes, “Quality of work” and “Total productivity”, had the greatest weights. Sensitivity analysis indicated that although some factors had stronger influence in the outer nodes of the network, there was relatively equal influence of the factors leading directly into the target node. Despite the differences in the node weights provided by our supra-experts, there was good agreement among assessments of our hypothetical experts that accurately reflected differences we had specified. This systems approach provides a way of assessing the overall level of expertise of individuals, accounting for multiple contributory factors, and their interactions. Our approach is adaptable to other situations where it is desirable to understand components of expertise.

## Introduction

The use of expert knowledge is gaining currency in scientific research and decision-making (O'Hagan 1998; Ayyub 2001; O'Hagan et al. 2006). Consequently, expert knowledge is being increasingly used in a diverse range of disciplines where more traditional types of empirical data are insufficient to address particular issues in a specific context and/or in a timely manner. These discipline areas include landscape ecology (Low Choy et al. 2009), conservation and management of threatened and endangered species (Campbell 2002; Smith et al. 2007; Murray et al. 2009; O'Leary et al. 2009; James et al. 2010; Johnson et al. 2010; Martin et al. 2012), environmental risk (Hamilton et al. 2007; Hoelzer et al. 2012; Johnson et al. 2013a,b2013b), meteorology (Risk Management Services 2006), climate change (Risbey 2008), health and medicine (Knol et al. 2010; Waterhouse and Johnson 2012), knowledge engineering (Kendal and Creen 2007), information technology systems (Franke et al. 2012) and industry (Yu 2002). Central to the use of expert knowledge in these situations are the subjective probabilities associated with the elicitation of expert knowledge (Cox 2000; O'Hagan et al. 2006). Such use of subjective probability has been supported from theoretical and practical perspectives, particularly in a Bayesian statistical framework (e.g., Low Choy et al. 2009; Oakley and O'Hagan 2010; Fisher et al. 2012).

Situations in which expert knowledge is required can arise for many reasons: a situation may be novel because environmental, social, and/or economic conditions have, or are projected, to change; priorities of stakeholders may shift through time because of the emergence of new information, or situations; decisions or risk assessments might need to occur quickly, precluding further information gathering; or, the required empirical information is simply unknown, or unknowable, for the foreseeable future. Because of the broad utility of expert knowledge, its growing popularity as a research tool and recognition that the credibility of elicited knowledge is ultimately determined by the rigor of the design and execution of elicitation methods (Low Choy et al. 2009; Kuhnert et al. 2010; Low-Choy et al. 2012), considerable recent effort has focused on developing formal methods for eliciting expert knowledge and applying expert judgement derived from this knowledge (Loveridge 2002; The Royal Society of Canada 2004; Refsgaard et al. 2007; Fisher et al. 2012). For example, the best ways to choose experts for particular applications and elicit their knowledge while attempting to control for potential cognitive, motivational, and behavioral biases are becoming well established in some fields (reviewed by Drescher et al. 2012). While consensus appears to be emerging regarding many aspects of the practice of expert elicitation, further research is required in many others.

Central to the practice of eliciting and applying expert judgement is what constitutes an expert, and once defined as an expert, how robust assessments of degrees of expertise can be achieved and applied in particular situations. An expert is commonly defined as someone with comprehensive and authoritative knowledge in a particular area not possessed by most people. Expertise, in turn, can be substantive, where knowledge is of a particular domain usually gained through training and professional practice, normative, the ability to communicate judgements clearly and accurately, and adaptive, the ability to adapt or extrapolate to new situations (Martin et al. 2012). Expertise can also be local and/or general, existing at different spatial and temporal scales or different functional levels (McBride et al. 2012). Expert knowledge, however, is unlikely ever to be completely accurate or certain, especially where experts are engaged because of the novelty of a situation and where empirical information is limited. Indeed, in the absence of appropriate training, level of expertise, either self-assessed or estimated from simple metrics, is not necessarily a good predictor of expert performance (McBride et al. 2012). Moreover, individual experts may not be expert in all aspects of a problem where expert knowledge is being applied. By carefully defining the expertise required for a particular application and the level of expertise of individual experts, more informed choices will be possible in choosing an appropriate sampling universe, whether it be a single expert, a range of experts elicited individually, larger groups of experts elicited using a Delphi-like process, or some combination of these.

Because of the limits to expert knowledge, it is common to elicit multiple experts possessing expertise in a range of disciplines, and multiple experts in the same disciplines, when addressing a particular issue (Martin et al. 2005). In such situations, expert judgements can be expected to vary (e.g., Campbell 2002; Martin et al. 2005; O'Leary et al. 2009) and combining and/or weighting (Burgman et al. 2011) opinions of multiple experts should provide better aggregate judgements. However, in some circumstances, such as policy decisions, it may be preferable to represent the diversity of expert judgements for effective and informed decision-making and planning, rather than presenting an aggregated unified position. In most cases though, individuals are chosen for elicitation exercises simply because they are deemed to be expert in some aspect of the problem of interest. Rarely is any attempt made to assess quantitatively degrees of expertise in ways that explicitly evaluate the many potential factors and their interactions that may contribute to an individual's total level of expertise (but see O'Leary et al. 2011 for a conceptual model of expertise).

The complexity of factors that contribute to expertise, their interactions, and the relative importance of these factors and interactions, all motivate a systems approach to describing an expert. Systems approaches are becoming widely used for modeling complex processes or concepts (Cowell et al. 1999). They provide a way of formally representing complexity and, where appropriate, quantifying the components of a system in order to obtain an overall probabilistic assessment of the final outcome. In a model of experts, the final outcome could be an estimate of an individual's expertise. A popular systems model is a Bayesian network in which various factors, and their interactions are depicted as a directed graph and then probabilistically quantified (Jensen and Nielsen 2007).

One way of quantifying a systems model is to adopt a supra-Bayesian approach. In the context of a BN for expertise, we define a supra-Bayesian or supra-expert (hereafter) as an independent expert who estimates a series of indicators of expertise for a group of individuals that act as the primary experts within a particular discipline. Beyond controlling for potential biases associated with self-assessment by experts of themselves, such a supra-Bayesian approach can be used to assess a large number of potential indicators of expertise in a BN that accounts for relationships among them. Where more than one supra-expert is available, combining and weighting their multiple opinions should also have similar advantages as combining expert judgements. In some situations where the use of expert knowledge is desirable, multiple experts with overlapping areas of expertise may not be available, and there may be no opportunity to calibrate experts using known values; such cases can arise, for example, in taxonomy (Fisher et al. 2012), medicine (EUCERD[Bibr b12]), and the construction of concept maps (Coffey et al. 2002). In such situations, it may be even more important to independently “calibrate” the responses of these individual experts using supra-experts to evaluate the expertise of primary experts.

Here, we report the development of a systems approach to defining expertise, using a Bayesian network model and a supra-Bayesian method for its quantification. For concreteness, we focus on the particular problem of assessing levels of expertise in estimating species richness, in this case, the global species richness of multicellular organisms on coral reefs and based on the conceptual model presented by O'Leary et al. (2011). Estimating the number of species either globally or by habitat is a significant problem in ecology, conservation, and resource management (e.g., Mora et al. 2011; Appeltans et al. 2012; Costello et al. 2013; Hamilton et al. 2013). Without adequate, baseline knowledge of the sizes of these species pools, it is impossible to know if species are being lost, or remedial action taken to conserve and manage them is being effective. Despite many attempts over the past decades to estimate total species richness at these large spatial scales, no agreement among these estimates has yet been forthcoming (Caley et al. unpublished, *cf*. Costello et al. 2013). This lack of convergence is perhaps not surprising, given that for many taxa, the taxonomy is very incomplete. Given the large numbers of species compared with the number of professional taxonomists, discovering, describing, and naming species, decades to centuries will be required to complete the task at current rates of description and depending on how many species actually inhabit Earth (Costello et al. 2013). As a consequence of the existence of this large number of unknown species, estimates of total species richness must rely on some form of statistical extrapolation, often from species discovery curves. Such extrapolation, though, is likely to be compromised for species discovery curves where sampling effort and success of individual collectors (Bebber et al. 2012) varies through time, and by the curvature of such relationships (Bebber et al. 2007). Unless a large proportion of a species pool has been discovered, estimates from extrapolation along such curves will likely be highly uncertain (Bebber et al. 2007).

Elsewhere, we have presented a method for estimating total species richness on tropical coral reefs based on the elicitation of expert judgement (Fisher et al. 2012). Our experts were professional taxonomists, and as part of a very knowledge-rich profession, they have much to offer in advancing understanding of total species richness (e.g., Appeltans et al. 2012). Because of the generally incomplete state of taxonomy and because coral reefs host a very considerable portion of all marine species (Knowlton et al. 2010; Plaisance et al. 2011), typically only one or a few taxonomists are available to elicit knowledge from regarding any particular coral reef taxon. This limited number of taxonomists precludes the use of some commonly used protocols for eliciting expert judgements such as broad-scale surveys and workshops. Similarly, the highly specialized nature of taxonomic knowledge demands methods that ensure maximum value is derived from the expert knowledge that is available. For example, where only a single taxonomist is available with knowledge of a particular taxon, it may be appropriate to weight the uncertainty of their estimates by their level of expertise. Alternatively, where two or more taxonomists can be elicited regarding the same taxon, weighting of both their uncertainty and best estimates by their level of expertise may be desirable. While potentially beneficial, to date, no methods that can simultaneously account for a large number of factors related to expertise have been reported.

## Material and Methods

We developed an explanatory systems model of levels of expertise in the form of a BN. As described above, in order to provide an explicit framework for the development of this model, the target group was chosen to be practicing professional taxonomists, and the outcome of interest was the estimation of their levels of expertise.

The BN was constructed in two stages: the specification of the BN structure and its probabilistic quantification. In the first stage, the BN structure was developed through a focus group convened with three eminent taxonomists Drs. A. Hosie, P. Doughty, and M. Harvey of the Western Australian Museum, and three of the authors of this study (MJC, RF, and RO). This group was selected to represent diversity of ecological, academic, and field expertise. A Delphi facilitation process was used to elicit from the taxonomists the following information (MacMillan and Marshall 2006). First, we established the factors considered to be important contributors to “level of expertise”. Agreement was reached on the definition of each factor. All factors were then scaled between 0 and 10 with 10 indicating the most favorable outcome with respect to level of expertise (Table 1). Second, we developed a graphical model of the relationships among these factors, with nodes representing factors and unidirectional arrows representing relationships between them. The only constraint imposed on the graphical representation was that it be acyclic (i.e., no loops within the network), in order to preserve the probabilistic integrity of the BN.

**Table 1:** Descriptions of criteria and scoring scheme used in assessing the level of expertise of taxonomists.

Criterion	Scoring (0–10)
1) Publishes in reputable peer-reviewed international journals	0 = never 10 = always
2) Taxonomic descriptions comprehensive and high quality	0 = never 10 = always
3) Taxonomic descriptions subsequently synonymized	0 = always 10 = never
4) Adheres closely to international standards of taxonomic nomenclature	0 = never 10 = always
5) Overall quality of taxonomic work	0 = the world's worst 10 = the world's best
6) Number of new taxonomic descriptions and redescriptions published	0 = none 10 = the most prolific worldwide
7) Research outputs beyond taxonomic descriptions such as checklists, monographs, and interactive keys.	0 = none 10 = the most prolific worldwide
8) Career-to-date, total productivity across all categories relative to others in this taxonomic community	0 = none 10 = the most prolific worldwide
9) Total contribution to coral reef taxonomy across all taxa	0 = none 10 = the most prolific worldwide
10) Possesses and employs a wide range of statistical and phylogenetic analytical skills	0 = applies no such skills 10 = the most skillful worldwide
11) Collects and/or analyses genetic data and applies it to taxonomic descriptions and/or revisions	0 = never 10 = always
12) Collects and/or analyses morphological data and applies it to taxonomic descriptions and/or revisions	0 = never 10 = always
13) Overall methodological breadth	0 = none 10 = applies all methods currently available
14) Breadth of ecosystems studied (can include sampling or analysis of samples/data acquired by others)	0 = Coral reefs only 10 = all ecosystems worldwide that host their taxa of interest
15) Breadth of habitats studied (can include sampling or analysis of samples/data acquired by others)	0 = a single habitat 10 = all habitats that host their taxa of interest
16) Breadth of taxa studied (can include sampling or analysis of samples/data acquired by others)	0 = none 10 = greatest breadth of any coral reef taxonomist worldwide
17) Geographic reach of their studies (can include sampling or analysis of samples/data acquired by others)	0 = a single geographic region 10 = all geographic regions hosting their taxa of interest
18) Overall sampling breadth	0 = narrowest of all taxonomists 10 = broadest of all taxonomists
19) Grant success	0 = least successful worldwide 10 = most successful worldwide
20) Prizes, accolades	0 = the fewest worldwide 10 = the most worldwide
21) Professional pedigree	0 = entirely self-taught 10 = trained by the best
22) Valued collaborator	0 = never sought as a collaborator 10 = collaborator in the greatest demand worldwide
23) Training and mentoring	0 = has never trained or mentored a junior taxonomist 10 = trained or mentored more junior taxonomists than anyone else
24) Professional standing as a taxonomist	0 = the world's least respected taxonomist 10 = the world's most respected taxonomist
25) Overall status as a taxonomic expert considering all these criteria together	0 = the very worst 10 = the world's very best

In the second stage and in accordance with the recommended BN development cycle (Johnson and Mengersen 2012), four senior professional taxonomists (J. Hooper, Queensland Museum, G. Rouse, Scripps Institute of Oceanography, P. Bouchet, Muséum national d'Histoire naturelle, and T. Gosliner, California Academy of Sciences), acting as “supra-experts”, were asked to independently assign values between 0 and 1 to each factor, conditional on the factors impacting on it (i.e., its parent nodes, indicated by directed arrows feeding into it in the graphical model). This value represented the relative importance, or weight, attributed to that factor by the supra-expert. The associated conditional probability table (CPT) was then computed as a rescaled linear combination of the weights ascribed to the parent nodes. For example, for a node C with two parent nodes A and B, where all nodes are categorized as High (H) (i.e., value = 1) and Low (L) (i.e., value = 0), and where weights *w*_A_ and *w*_B_ denote the weights ascribed to nodes A and B, respectively, the CPT was calculated as shown in Table 2. Further commentary on this approach is provided in the Discussion.

**Table 2:** Illustration of using node weights (*w*_A_, *w*_B_) to quantify the conditional probability table for an internal node C, based on input nodes A and B.

Parent node	Node value			
A	H	H	L	L
B	H	L	H	L

Child node	Weighting formula	Conditional probability	Weighting formula	Conditional probability

*Pr(C=H / A,B)*	*w*_A_/(*w*_A_ + *w*_B_)	1	*w*_B_/( *w*_A_ + *w*_B_)	0
*Pr(C=L / A,B)*	1−*w*_A_/( *w*_A_ + *w*_B_)	0	1−w_B_/(*w*_A_ + *w*_B_)	1

Four separate BNs were constructed using the structure agreed in Stage 1 and the CPTs obtained for each supra-expert in Stage 2.

The BN was validated using criteria adapted from Pitchforth and Mengersen (2013) (Table S1). First, the nominological, face, content, convergent, and discriminant validities of the network were assessed by presenting the network structure to our supra-experts in their capacities as professional taxonomists when they provided us with node weights. At these times, no concerns about these aspects of validity were raised. Second, concurrent validity was assessed by the statistical analysts when the model was quantified. Third, predictive validity was assessed by computing the network probabilities for a set of hypothetical experts representing career paths of early, mid- and late-career taxonomists (Table 3); the results obtained for sub-networks of the model and the overall network were then assessed for consistency and comparative ranking within and across the hypothetical subjects and the four supra-experts. Finally, overall model validity, comprising a review of all seven validity checks, was independently confirmed by presenting the network to a wider group of experts in Bayesian statistics and systems modeling outside taxonomy.

**Table 3:** Hypothetical experts and associated scores for each factor in the Bayesian network (BN). Abbreviated criteria are used here. See Table 1 and text for further explanation of these criteria and how they were scored. Scores are provided here for primary nodes only. Values for higher-order nodes (indicated by –) and used in Figure 4 were calculated conditional on the values presented here and weights of their immediately subordinate nodes provided by the supra-experts (Fig. 2).

	Characteristics of hypothetical taxonomists						
Criterion	Late career, world's best	Late career, well respected, specialist on particular group(s)	Late career, does poor-quality work	Mid-career, respected high achiever	Mid carrier, respected, researches beyond taxonomy	Early-career researcher, respected, getting established	Ph.D. student in taxonomy
1) Publishes in reputable journals	10	9	5	10	8	9	9
2) Taxonomic descriptions high quality	10	9	5	10	8	9	9
3) Descriptions synonymized	10	9	3	9	7	7	5
4) Adherence to rules of nomenclature	10	10	5	10	8	9	9
5) Quality of work	–	–	–	–	–	–	–
6) Number of taxonomic descriptions	10	9	7	7	5	4	0
7) Research outputs (other)	10	9	7	7	5	2	0
8) Total productivity	–	–	–	–	–	–	–
9) Total contribution	–	–	–	–	–	–	–
10) Analytical skills	10	7	3	5	3	6	5
11) Genetics	10	4	1	7	3	7	5
12) Morphology	10	10	9	7	8	5	5
13) Methodological breadth	–	–	–	–	–	–	–
14) Ecosystem breadth	10	10	9	7	5	6	2
15) Habitat breadth	10	10	9	7	5	7	2
16) Taxonomic breadth	10	8	9	7	5	5	1
17) Geographic reach	10	9	9	7	5	6	2
18) Sampling breadth	–	–	–	–	–	–	–
19) Grant success	10	8	6	6	3	3	0
20) Prizes, accolades	10	8	5	5	3	1	0
21) Professional pedigree	10	5	5	8	5	5	5
22) Valued collaborator	10	8	3	6	3	3	0
23) Training and mentoring	10	7	3	5	3	2	0
24) Professional standing	–	–	–	–	–	–	–
25) Taxonomic expert	–	–	–	–	–	–	–

The completed BNs were used to conduct three main evaluations. First, the supra-experts were compared with respect to the scores that they assigned to the set of nodes. Second, the BNs were interrogated to identify the relative importance of the factors in characterizing level of expertise. This was used to develop a “profile of expertise” and to address the primary question, “what makes a ‘good’ expert?” Third, hypothetical experts were compared with respect to their scores obtained from each of the four BN models. An aggregate score and associated variance were obtained for each of these hypothetical experts by combining the scores assigned to them based on the node weights provided by each of the supra-experts.

## Results

### BN structure

**Figure 1:**
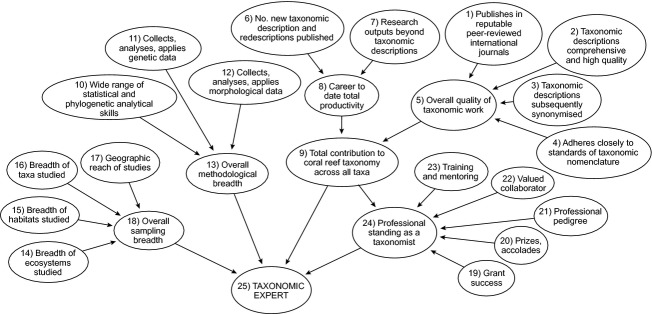
Structure of an expert-derived Bayesian network illustrating acyclic relationships among factors describing the level of expertise of professional taxonomists. See Table 1 and text for descriptions of these factors and how they were scored.

The factors that were considered to be important by the focus group in the evaluation of an “expert” are shown in Table 1. The corresponding BN structure that was developed to describe the interactions of these factors is depicted in Fig. 1. The resulting BN consisted of 18 primary nodes feeding through between one and three higher-order nodes before converging on the target node (Taxonomic Expert). All links between nodes, except one, linked a node at a lower level to a single node at the immediately higher level in the hierarchy rendering a quite simple net structure. The primary nodes grouped at higher nodes that defined overall sampling and methodological breadth, quality and quantity of contributions to taxonomy, and the expert's overall standing as a professional taxonomist.

### Node weights

**Figure 2:**
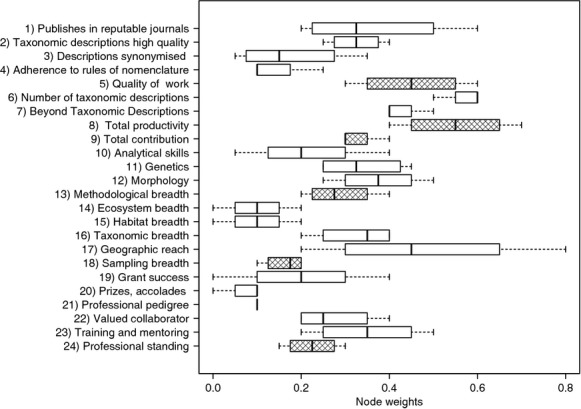
Node weights provided by four independent supra-experts. Hatched boxes indicate higher-order nodes.

The median weights provided by the supra-experts ranged between 0.1 and 0.6 (Fig. 2). For some nodes, there was very strong consistency among supra-experts (e.g., the importance of professional pedigree, and the breadth of ecosystems and habitats sampled during a career), whereas other nodes (e.g., the importance of geographic reach of sampling) ranged from having little importance (weight = 0.2) to the greatest importance ascribed to any node (weight = 0.8). Of the higher-order nodes, “Quality of work” and “Total productivity” were given greatest weight.

### Importance of factors for describing an expert

The BN depicted in Fig. 1 was quantified using the node weights provided by the four supra-experts. The four BNs were then interrogated to identify the relative importance of the factors in characterizing level of expertise. This was used to develop a “profile of expertise” and to address the primary question, “what makes a ‘good’ expert?”

**Figure 3:**
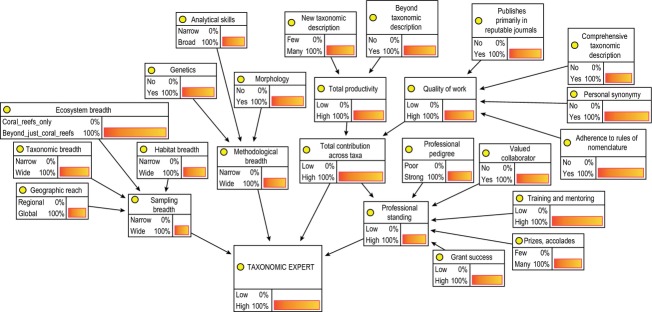
Quantified Bayesian network (BN) for our most expert hypothetical subject, based on the judgement of supra-expert 1 (JH). Thicker arrows indicate more influential factors in the BN.

Sensitivity analysis of the BN indicated that although some factors had stronger influence in the outer nodes of the network, there was relatively equal influence of the factors leading directly into the target node (Taxonomic Expert). This is illustrated in Figure 3, which depicts the quantified BN for hypothetical subject 1 (Best worldwide), based on the judgement of one supra-expert.

### Comparison of hypothetical experts

**Figure 4:**
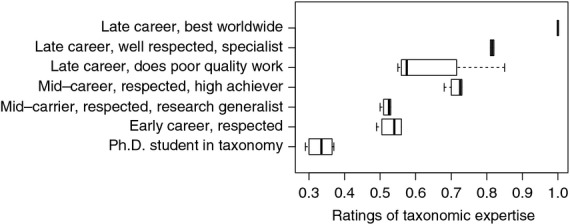
Ratings of the expertise of seven hypothetical categories of taxonomist.

Probabilities were assigned to the hypothetical experts that they possess a “High” level of each of the features in the BN (Table 3). These probabilities were entered into the separate BNs for each of the four supra-experts. The resultant probabilities of a “High” level of taxonomic expertise for each of the hypothetical subjects, obtained from each of the four BNs, are depicted in Figure 4.

Despite some differences in node weights provided by the supra-experts, there was a remarkably high level of agreement in the overall assessments of expertise when these weights were applied to the assessment of our hypothetical experts. Our model was also able to capture expected differences specified for the hypothetical experts. For example, assessed median expertise increased as expected from the Ph.D. student category, through to the top category, “Late career, best worldwide”. The respected early-career taxonomist ranked favorably with the mid-career taxonomist that does not concentrate solely on taxonomy. Both these categories of expert were outranked by the high-achieving, mid-career taxonomist. The late-career, well-respected taxonomist that has spent most of their career doing taxonomy out-ranked all three. Ratings of the late-career taxonomist that does poor-quality work were the most variable. Gratifyingly though, the median expertise score for this category of taxonomist was more similar to the mid-career categories than to the other late-career categories.

## Discussion

The increasing use of expert knowledge in many disciplines has led to a very rapid increase in research and understanding of how best to capitalize on this very valuable resource. Expert knowledge can be difficult to capture comprehensively and archive appropriately as it resides with the experts themselves and is therefore subjective in nature as is the uncertainty that surrounds it (Cox 2000; O'Hagan et al. 2006). Consequently, expert knowledge is not amenable to more traditional methods of collecting and analyzing information (Martin et al. 2012), but these subjective probabilities can be explicitly accommodated in Bayesian analyses (Low Choy et al. 2009; Oakley and O'Hagan 2010; Fisher et al. 2012) as developed here. Moreover, the acquisition of such knowledge by experts typically represents a substantial investment of resources over long periods. It is important therefore to understand how best to capture and use this valuable information. One particularly important gap in understanding how best to use expert knowledge is how to account for the many potential factors, and their interactions, that determines an individual's total level of expertise. By better understanding the total expertise of individuals, more robust comparisons of expertise among individuals can be supported.

The importance of assessing level of expertise has been recognized in earlier research that has applied various techniques to rank experts (e.g., McBride et al. 2012). In contrast to these previous studies, we have considered the issue of expertise from a systems perspective and developed an explanatory Bayesian network model that allows explicit and simultaneous evaluation of many possible factors and their interactions in a quantitative framework. These evaluations were used to assess the implications for choosing experts and using their expert knowledge. Our approach also presents a number of additional advantages. We capitalized on the experience and expert knowledge of disciplinary practitioners in a focus-group setting in developing the BN structure. This structure simultaneously captured a large number of factors contributing to expertise in this particular situation, and the most direct and likely relationships between them.

The provision of node weights for the BN by a second set of expert taxonomists, our supra-experts, allowed us to assess variation in the assignment of importance to these factors among taxonomists. Their expert judgements regarding relative importance were very consistent for some factors and quite divergent for others. For the primary nodes, median weights and the corresponding variation differed considerably both among supra-experts and among nodes (Fig. 2). This pattern was also evident for groups of primary nodes feeding into the same higher-order node (e.g., Fig. 2: nodes 1–4, nodes 14–17, nodes 19–23). For example, “Adheres closely to standards of taxonomic nomenclature” was not given much weight, presumably because most practising taxonomists adhere to these rules and the rules are enforced to a large extent during peer review of taxonomic descriptions before they are published. Similarly, “Professional pedigree” was accorded little importance. At higher levels in the hierarchy beyond the primary nodes, a great deal of this variation disappeared and “Quality of work” and “Total productivity” emerged as the child nodes of greatest weight in defining taxonomic expertise (Fig. 2). In contrast, “Professional standing” was given less weight apparently because most of the taxonomic expertise of an individual had already been captured by the node, “Total contributions”.

Irrespective of differences in node weights assigned by each of the supra-experts, application of these node weights to hypothetical profiles of seven categories of taxonomist revealed strong consistency in the rankings of the supra-experts, both within and among categories that aligned with the expectation that expertise should increase from early training to late in one's career and that the level of expertise obtained will be determined in part by the opportunities presented and/or taken to concentrate solely on the pursuit of a particular discipline. The observation that while node weights may vary among supra-experts, very similar overall rankings can be obtained, in this case of our hypothetical experts, reveals a convenient and potentially powerful flexibility of this approach.

During the elicitation of node weights, it was evident that different supra-experts attributed greater weight to some parts of the BN than others, in essence, choosing different pathways through the model to achieve similar ends (M. J. Caley, pers. obs.). For example, where one supra-expert might weight heavily the importance of professional standing, assuming its attainment is some function of career accomplishments, others would weight heavily direct contributions to taxonomy such as naming many species, while assuming that professional standing would derive from this productivity, and assigned this factor little additional weight.

The one category of hypothetical taxonomist not scored consistently by the BN based on the node weights of the supra-experts was the late-career taxonomist that does poor-quality work, and who is therefore, not particularly well respected. The source(s) of this discrepancy were not pursued, but could be many. For example, different supra-experts may deem different aspects of a person's taxonomy to be poor quality. Alternatively, some supra-experts may allow an assessment of poor quality for one factor to bias their assessment of other factors. Most likely, however, there are many ways to do poor-quality work, and such variation was not adequately captured by our model. This proposition could be tested using a set of profiles for hypothetical taxonomists that do poor-quality work that better captures possible sources of this variation to see if more consistent ratings can be achieved.

Because this BN quantifies the contribution of many factors and their interactions that may determine an individual's expertise, it provides new opportunities for capturing and using expert knowledge. For example, the model provides uncertainty around ratings of expertise, and where expertise is evaluated by multiple supra-experts, ratings of expertise can be combined and uncertainty explored further. Where more than one expert is available to provide opinions on a particular item, their opinions and the uncertainty around them might be weighted by ratings of expertise. Where only one expert is available, it may still be useful to weight their uncertainty. There may also be utility in using overall expertise ratings in choosing expert panels, or alternatively, to assemble panels where the expertise represented by the panel is maximized by selecting individuals that score very highly on some factors but not others, whereby, at least one expert is highly ranked for every factor. Where experts can be rated prior to elicitation, training for the task might be designed to emphasize areas where expertise is least and/or inherent biases are greatest. Lastly, by understanding the strengths and weaknesses within and among experts, it may be possible to provide training whereby expertise is improved so that performance is maximized on specific tasks and to better understand the importance of differences in opinions of the experts.

Although the approach we present here has much to recommend it, it has technical limitations as well. The method of combining node weights to derive the CPTs for the BN is confined to nodes with only two categories, although the approach can be extended for more categories. Moreover, it does not allow for full quantification of all possible interactions between factors contributing to a child node, only those that were nominated by experts as being sufficiently important to be incorporated in the model's structure. Nonetheless, we deemed it to be a robust method of elicitation that avoided many of the potential biases inherent in eliciting complex probabilities from nonstatistically trained respondents (Low Choy et al. 2009). Finally, instead of allocating probabilities of 0 (indicating no chance) or 1 (indicating absolute certainty) in the BN tables, it may be more appropriate to set these as 0.01 or 0.99 to allow for a small amount of uncertainty in the outcome or tolerance in the definition of the corresponding factor. The substantive inferences from this assessment were unchanged so the results are not reported here. The structure of the model could also be extended by the addition of additional nodes to account for biases, should they be of concern in other situations. It could also be extended by the inclusion of additional submodels if it was desirable to account for different degrees of expertise by different groups of experts related to different parts of the problem (e.g., Johnson et al. 2013a,b2013b). Based on the information available in our study, there was no evidence that including such nodes or submodels into our model would have improved the inferences that could be drawn from it.

In summary, the systems modeling approach reported here appears to quantify well taxonomic expertise by modeling many interacting factors in an explicit relational framework. While our study involves analyses of taxonomic expertise, our primary goal was neither to provide an assessment nor a tool for its specific assessment. Our intent, instead, was to develop a method for assessing degrees of expertise applicable to situations where expert opinions are available from many individuals collected independently (i.e., not during group elicitation such as during a Delphi-style facilitation process). While individual-based elicitation is likely to impose additional overheads over group-based approaches, some of the biases imposed by group dynamics (O'Hagan et al. 2006) may be better controlled. Such individual-based elicitations are likely to allow for better use of expert knowledge in a variety of situations. Knowing whether our expectation here is borne out will require explicit comparisons of this and other approaches as they are developed.

## Supporting Information

Table S1 Validity assessment for expert elicited Bayesian Networks.
