# Supplementary material for: What is an expert? A systems perspective on expertise
Source: Ecol Evol. 2013 Dec 26;4(3):231–42. doi: 10.1002/ece3.926 (PMC3925425; doi:10.1002/ece3.926)
Supplement: Table S1 — Validity assessment for expert elicited Bayesian Networks. [file ece30004-0231-sd1.docx]

**Table S1.** Validity assessment for expert elicited Bayesian Networks*

| Type of Validity | Question |
| --- | --- |
| Nominological | Does the BN model fit within an appropriate context in the literature? |
|  | Which themes and ideas are nomologically adjacent to the BN model, and which are nomologically distant? |
| Face | Does the model structure (the number of nodes, node labels and arcs between them) look the same as the experts and/or literature predict? |
|  | Is each node of the network discretised into sets that reflect expert knowledge? |
|  | Are the parameters of each node similar to what the experts would expect? |
| Content | Does the model structure contain all and only the factors and relationships relevant to the model output? |
|  | Does each node of the network contain all and only the relevant states the node can possibly adopt? |
|  | Are the discrete states of the nodes dimensionally consistent? |
|  | Do the parameters of the input nodes and CPT reflect all the known possibilities from expert knowledge and domain literature? |
| Concurrent | Does the model structure or sub-networks act identically to a network or sub network modelling a theoretically related construct? |
|  | In identical sub networks, are the included factors discretised in the same way as the comparison model? |
|  | Do the parameters of the input nodes and CPTs in networks of interest match the parameters of the sub network in the comparison model? |
| Convergent | How similar is the model structure to other models that are nominologically proximal, i.e., intended to describe a similar system? |
|  | How similar is the discretisation of each node to the discretisation of nodes that are nomologically proximal independent of their network domain? |
|  | Are the parameters of nodes that have analogues in comparison to models assigned similar conditional probabilities? |
| Discriminant | How different is the model structure to other models that are nomologically distal, i.e. that should be describing a different system? |
|  | How different is the discretisation of each node to the discretisation of nodes that are nomologically distal independent of their network domain? |
|  | Are the parameters of nodes in the comparison models that have oppositional definitions to the node in question parameterised differently? |
| Predictive | Is the model behaviour predictive of the behaviour of the system being modelled? |
|  | Once simulations have been run, are the output states of individual nodes predictive of aspects in the comparison models? |
|  | Is the model sensitive to any particular findings or parameters to which the system would also be sensitive? |
|  | Are there qualitative features of the model behaviour that can be observed in the system being modelled? |
|  | Does the model including its component relationships predict extreme model behaviour under extreme conditions? |

*adapted from Pitchforth and Mengersen (2013)
